# Erholungs-Beanspruchungs-Zustand im Rettungsdienst während der ersten beiden Wellen der SARS-CoV-2-Pandemie

**DOI:** 10.1007/s10049-022-01102-z

**Published:** 2022-11-29

**Authors:** Heiko Schumann, Julia Botscharow, Beatrice Thielmann, Irina Böckelmann

**Affiliations:** Bereich Arbeitsmedizin, Medizinische Fakultät, Otto-von-Guericke-Universität Magdeburg, Leipziger Str. 44, 39120 Magdeburg, Deutschland

**Keywords:** Psychische Belastung, Regeneration, Medizinisches Personal, Gefährdungsbeurteilung, COVID-19-Pandemie, Mental stress, Regeneration, Medical personnel, Risk assessment, COVID-19 pandemic

## Abstract

**Hintergrund:**

Die Balance zwischen Belastungsempfinden und Erholung der Einsatzkräfte im Rettungsdienst während des Diensts und in der Freizeit ist ein wesentlicher Indikator für die psychische und physische Gesundheit. Sie gewährleistet Erfolg in der notfallmedizinischen Versorgung und ist gleichzeitig auch ein gesundheitspolitischer, ökonomischer Faktor für jede Organisation. Das Ziel der Studie war es, den Erholungs-Beanspruchungs-Zustand des Rettungsdienstpersonals während der ersten und zweiten Welle der SARS-CoV-2-Pandemie zu analysieren.

**Material und Methoden:**

Die quantitative Querschnittstudie umfasst 1936 Datensätze von Einsatzkräften (334 Frauen und 1602 Männer, Durchschnittsalter 34,9 ± 10,5 Jahre). Die Onlinebefragung während der SARS-CoV-2-Pandemie erfolgte während der ersten Welle 2020 (t1) und der zweiten Welle 2021 (t2). Es wurde die Kurzform EBF-24/A (Testform S2) des Erholungs-Belastungs-Fragebogens (EBF) nach Kallus angewendet.

**Ergebnisse:**

Im Verlauf der beiden Erhebungsphasen nahm die Dimension Beanspruchung signifikant zu (t1: 2,52 ± 0,98 und t2: 2,88 ± 1,04 Punkte, *p* < 0,001) und die Erholung signifikant ab (t1: 2,98 ± 0,90 und t2: 2,64 ± 0,89 Punkte, *p* < 0,001). Ähnliches bot sich auch für die dazugehörigen Subskalen mit Ausnahme der Subskala „erholsamer Schlaf“ der Dimension Erholung (t1: 2,81 ± 1,36 und t2: 2,72 ± 1,36 Punkte).

**Schlussfolgerung:**

Die seit Anfang 2020 bestehende SARS-CoV-2-Pandemie verdeutlicht, dass das Belastungserleben von der ersten zur zweiten Welle zugenommen und das Empfinden der Erholung für Einsatzkräfte im Rettungsdienst abgenommen hat. Die Studie bietet eine Einordnung zur gegenwärtigen Situation des Erholungs-Beanspruchungs-Zustands im deutschen Rettungsdienst und erlaubt Prognosen über Leistung und Gesundheit in Pandemiesituationen. Dabei ist davon auszugehen, dass die Verschlechterung des Ist-Zustands nicht einzig nur aus dem Arbeitskontext resultiert, sondern gleichzeitig ein Spiegelbild der Ressourcenausstattung darstellt, die sich aus der Gesamtheit der Einflüsse des Individuums ableitet.

## Abstract (en)

**Background:**

The balance between stress perception and recovery of emergency medical service personnel during service and leisure time is an essential indicator of mental and physical health. It ensures success in emergency medical care and is also a health policy and economic factor for any organization. The aim of the study was to evaluate the recovery stress level of paramedics in the first and second waves of the SARS-CoV‑2 pandemic.

**Materials and methods:**

The quantitative cross-sectional study included 1936 records of responders (334 women and 1602 men, mean age 34.9 ± 10.5 years). The online survey during the SARS-CoV‑2 pandemic was performed during the first wave in 2020 and the second wave in 2021. The short form EBF-24/A (test version S2) of the recovery stress questionnaire according to Kallus was analyzed.

**Results:**

During the course of the two survey phases, the dimensions stress increased significantly (t1: 2.52 ± 0.98 and t2: 2.88 ± 1.04 points, *p* < 0.001) and recovery decreased significantly (t1: 2.98 ± 0.90 and t2: 2.64 ± 0.89 points, *p* < 0.001). Similar results were found for the corresponding subscales with the exception of the subscale “restful sleep” of the dimension recovery (t1: 2.81 ± 1.36 and t2: 2.72 ± 1.36 points).

**Conclusion:**

During the SARS-CoV‑2 pandemic, which has been ongoing since the beginning of 2020, the stress experienced by emergency responders increased from the first to the second wave, while the perception of recovery for emergency responders decreased. The study presents the current situation of recovery stress in German emergency medical service employees and allows predictions about performance and health in pandemic situations.

## Hinführung zum Thema

Rettungsdienstkräfte sind sowohl im Einsatz als auch im Arbeitsalltag auf der Rettungswache während der SARS-CoV-2-Pandemie zusätzlichen physischen als auch psychischen Belastungen ausgesetzt [24]. Die Zunahme der Belastung ist für Erholungsbeeinträchtigung mitverantwortlich. Darüber hinaus ist zu konstatieren, dass die Voraussetzungen für Erholungsprozesse im Alltag selbst auch beeinträchtigt sein können (z. B. durch die Betreuung von Kindern, Homeschooling, Homeoffice der Lebenspartner, soziale Isolation; [26]). Diese Belastungen können auch Erholungsprozesse einschränken und langfristig Erkrankungen verursachen [19, 24].

## Hintergrund und Fragestellung

In dem Modell der Erholungs-Beanspruchungs-Bilanz [16] wird das Modell zum Stressgeschehen [14] mit psychophysischen Prozessen kombiniert. Dieses Modell ist die Grundlage der vorliegenden Arbeit, um die Fragestellung, wie sich die belastende Situation in der ersten und in der zweiten Welle der SARS-CoV-2-Pandemie auf die Momentaufnahme des Erholungs-Beanspruchungs-Zustands der Rettungsdienstkräfte auswirkt, zu beantworten.

Ausreichende Erholung ist eine von mehreren Ressourcen für den Umgang mit belasteten Situationen, die zu einer Abnahme von Arbeitsstress beiträgt. Gute Erholung verhindert die Entwicklung von Fehlbeanspruchungen und Krankheiten [10]. Erholungsprozesse haben dabei eine besonders große Bedeutung [16]. Stressoren können vor allem dann Krankheiten verursachen, wenn die Möglichkeiten der Kompensationsmechanismen ausgeschöpft sind und wenn Erholungsprozesse nicht in ausreichendem Maße stattfinden. Beanspruchung und Erholung beziehen sich wechselseitig aufeinander [2].

Um die sehr anspruchsvollen Tätigkeiten im Rettungsdienst ausführen zu können, ist die physische und psychische Gesundheit der Einsatzkräfte eine Grundvoraussetzung [8, 12, 13, 23, 28]. Im Arbeitsalltag ist das Rettungsdienstpersonal einer großen Anzahl von physikalischen, physischen, psychischen sowie sozialen und organisationalen Stressoren ausgesetzt [23].

Im Rahmen der SARS-CoV-2-Pandemie besteht die Gefahr der Überlastung des Gesundheitssystems [18]. Lockdowns mit Kontakt- und Ausgangsbeschränkungen sowie Quarantänemaßnahmen sind mit zusätzlichen Belastungen verbunden [22], insbesondere für vulnerable Gruppen wie bspw. das Gesundheitspersonal [20, 25].

Gerade in dieser pandemischen Lage verschärft sich das Belastungserleben für das Rettungsdienstpersonal stark [24]. Neben der körperlichen Belastung durch das Tragen von umfangreicher persönlicher Schutzausrüstung (PSA) führten in der ersten Pandemiewelle fehlende Forschungserkenntnisse zur Ansteckungsfähigkeit und zu Übertragungswegen einer neuen, bis dato noch unbekannten Infektion sowie zum COVID-19-Verlauf zu großen Unsicherheiten und zu einer überdurchschnittlichen psychischen Belastung [3, 6, 27]. In der zweiten Pandemiewelle wurden diese Belastungen zusätzlich durch die hohe Anzahl der Infektionen, die strengen Quarantäneregelungen und den anhaltenden Personalmangel verstärkt.

Die mit der Pandemielage verbundenen Stressfaktoren führten vermutlich zur Steigerung der psychischen Belastungen, u. a. durch Angst, Depressivität, posttraumatische Belastungssymptomatik und Schlafstörungen [9], die sich bei einer fortwährenden Pandemielage weiter verschärfen könnten [4]. Diese beruflichen Belastungen können die Erholungsprozesse beeinträchtigen. Außerdem ist gerade in der Zeit der ersten Pandemiewellen für viele Beschäftigte mit den im Haushalt lebenden Schulkindern eine neue Situation eingetreten, da aufgrund von Homeschooling und Ganztagsversorgung der Kinder die Erholungs- und Regenerationszeit reduziert war [27].

Die Studie von Schumann et al. (2021) erfragte die subjektive Arbeitsbelastung während der 2. SARS-CoV-2-Welle (N 1203). 91,1 % der Einsatzkräfte des Rettungsdienstes antworteten auf die Frage „Hat die SARS-CoV-2-Pandemie zu einer Zunahme Ihrer täglichen Arbeitsbelastung geführt?“ mit „stimme voll zu“ oder „stimme zu“, lediglich 4 % antworteten mit „stimme nicht zu“ oder „stimme überhaupt nicht zu“, die restlichen 4,8 % gaben keine Tendenz an und stimmten neutral [24]. 58,1 % der Rettungsdienstkräfte berichteten von sinkender Arbeitszufriedenheit während der zweiten Coronawelle. Erhöhter Arbeitsstress in der Pandemie ist mit einer potenziellen Gesundheitsgefährdung für das Personal im Rettungsdienst verbunden [3].

## Untersuchungsmethoden

Die Onlinebefragung ermittelte den Erholungs-Beanspruchungs-Zustand von Einsatzkräften im Rettungsdienst während der ersten beiden Wellen der SARS-CoV-2-Pandemie. Die Rekrutierung der Stichproben erfolgte im Zeitraum von Juni bis August 2020 (1. Welle) und im Zeitraum von Januar bis März 2021 (2. Welle) deutschlandweit über soziale Medien und die Zeitschrift *Rettungsdienst*. Die Teilnahme an der Befragung war freiwillig und anonym. Die Rücklaufquote konnte aufgrund der Art der Rekrutierung als Onlinebefragung nicht rekonstruiert werden. Ein positives Votum der Ethikkommission der Medizinischen Fakultät der Otto-von-Guericke-Universität Magdeburg lag für die Studie vor.

### Probanden

Insgesamt wurden 1936 Datensätze von Einsatzkräften im Rettungsdienst im Alter von 18 bis 64 Jahren ausgewertet. Während der ersten Welle konnten 805 Fragebögen (651 männlich, 154 weiblich) und während der zweiten Welle 1131 Fragebögen (951 männlich, 180 weiblich) berücksichtigt werden. Als Einschlusskriterium für die Studie wurde eine hauptberufliche Tätigkeit in Voll- oder Teilzeit im Rettungsdienst als Einsatzkraft definiert.

### Variablen

Neben soziodemografischen und berufsbezogenen Daten wurde für die Ermittlung des aktuellen Grads der Erholung und Beanspruchung die Kurzform Erholungs-Belastungs-Fragebogen (EBF) EBF-24/A (Testform S2) mit 24 Items nach [15] genutzt. Dabei wurden das körperliche und seelische Befinden sowie die Aktivität innerhalb der letzten drei Tage und Nächte erfragt. Die Beantwortung der Fragen erfolgte auf einer 7‑stufigen Skala von 0 („nie“) bis 6 („immerzu“). Anschließend wurden die übergeordneten Dimensionen „Beanspruchung“ und „Erholung“ aus den Rohwerten der 12 Subskalen berechnet (vgl. Tab. 1). Hierbei deuten hohe Werte auf eine starke Beanspruchung bzw. eine ausreichende Erholung hin.

**Table 1:** 

Beanspruchung	Erholung
Allgemeine Belastung – Niedergeschlagenheit	Erfolg – Leistungsfähigkeit
Emotionale Belastung	Erholung im sozialen Bereich
Soziale Spannungen	Körperliche Erholung
Ungelöste Konflikte – Erfolglosigkeit	Allgemeine Erholung – Wohlbefinden
Übermüdung – Zeitdruck	Erholsamer Schlaf
Energielosigkeit – Unkonzentriertheit	
Körperliche Beschwerden	

Der angegebene akzeptable Bereich des Beanspruchungszustands liegt zwischen 0 = nie und 2 = manchmal und der des Erholungszustands liegt zwischen 4 = oft und 6 = immerzu [23]

### Datenanalyse

Die Daten wurden nach der Übertragung unter Anwendung des Psychodiagnostiksystems „Wiener Testsystem“ (Fa. Schuhfried, Mödling, Österreich) und des Statistikprogramms SPSS 26® (IBM, NEW York, USA) für Windows ausgewertet.

Entsprechend der Verteilungsform waren sowohl parametrische als auch nichtparametrische Tests erforderlich. Dabei wurde der t‑Test bei normalverteilten Variablen und der Mann-Whitney-U-Test bei nicht normalverteilten Variablen angewendet. Außerdem wurden für die statistische Auswertung Kreuztabellen mit dem Chi^2^-Test genutzt.

## Ergebnisse

### Soziodemografische Daten

**Figure 1:**
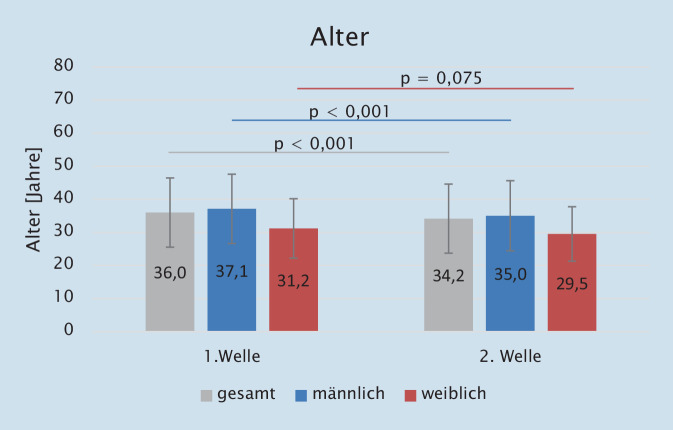

An der Studie nahmen insgesamt 1936 Rettungsdienstkräfte im Durchschnittsalter von 34,9 ± 10,5 Jahren teil (1. Welle: 36,0 ± 10,5 Jahre; 2. Welle: 34,2 ± 10,5 Jahre; *p* < 0,001). Abb. 1 veranschaulicht, dass das Alter der Männer bei der 1. Welle signifikant höher lag als bei der 2. Welle (p_U‑Test_ < 0,001). Die Altersunterschiede der Frauen bei den beiden Befragungen waren zwischen der 1. und 2. Welle nicht signifikant (p_U‑Test_ = 0,075).

Die Geschlechterverteilung der Stichproben bei den beiden Befragungen (19,1 % Frauen zu 80,9 % Männer in der 1. Welle und 15,9 % Frauen zu 84,1 % Männer in der 2. Welle) war statistisch nicht signifikant (*p* = 0,065). Der Anteil der Männer ist deutlich höher als der Anteil der Frauen (Tab. 2). Das Alter der Männer lag bei den beiden Befragungen signifikant höher (*p* < 0,001) als das der Frauen (1. Welle: 37,1 ± 10,5 Jahre vs. 31,2 ± 9,0 Jahre; 2. Welle: 35,0 ± 10,6 Jahre vs. 29,5 ± 8,2 Jahre).

**Table 2:** 

	1. Welle	2. Welle	Gesamt	*p*_χ_^2^_nach Pearson_
Männlich	651 (80,9 %)	951 (84,1 %)	1602 (82,7 %)	0,065
Weiblich	154 (19,1 %)	180 (15,9 %)	334 (17,3 %)	
Gesamt	805 (100 %)	1131 (100 %)	1936 (100 %)	

### Erholungs-Beanspruchungs-Zustand

Die interne Konsistenz Cronbachs alpha der Hauptskala Beanspruchung (α = 0,904) war als exzellent und der Hauptskala Erholung als hoch (α = 0,833) zu interpretieren. Damit ist die Reliabilität des Fragebogens aus statistischer Sicht gegeben und auswertbar.

Der Erholungszustand der Rettungsdienstkräfte unterschied sich in den beiden Wellen voneinander. Tab. 3 zeigt, dass die Mittelwerte der Dimension „Beanspruchung“, die sich aus den dazugehörigen Subskalen der Belastungen zusammensetzt, in der zweiten Welle höher waren (*p* < 0,001) und zugleich die Dimension „Erholung“, alle Subskalen eingeschlossen, geringer ausgeprägt war (< 0,001), was auf eine schlechtere Erholung hindeutet. Mit Ausnahme der Subskala „erholsamer Schlaf“ traten zwischen den Ergebnissen der beiden Wellen hoch signifikante Unterschiede auf. Bei dem Geschlechtergruppenvergleich findet man zu beiden Untersuchungszeitpunkten, dass die Frauen signifikant höhere Beanspruchung (*p* < 0,001) hatten. Während der 1. und der 2. Welle unterscheidet sich die Erholung der beiden Geschlechtergruppen nicht signifikant voneinander (*p* = 0,207 bzw. 0,374). Bei der ersten Welle bestehen die signifikanten Unterschiede zwischen den Geschlechtern in den Subskalen „allgemeine Belastung – Niedergeschlagenheit“, „emotionale Belastung“, „Übermüdung – Zeitdruck“, „Energielosigkeit – Unkonzentriertheit“ und „körperliche Beschwerden“. Die Frauen waren höher beansprucht als die Männer. Bei der zweiten Welle kamen noch „hohe soziale Spannungen“ bei Frauen (*p* = 0,049) dazu, jedoch war Energielosigkeit – Unkonzentriertheit in den Geschlechtergruppen statistisch vergleichbar. Die körperliche Erholung zeigt bei der ersten Welle größere signifikante Differenzen zwischen den Männern und Frauen als in der zweiten Welle, wobei die Frauen eine schlechtere körperliche Erholung hatten.

**Table 3:** 

EBF-Merkmal	1. Welle				2. Welle				*p*_Mann-Whitney-U-Test_ _1. vs. 2._
	Median (Min-Max)				Median (Min-Max)				
	Gesamt *n* = 805	Männer (M) *n* = 651	Frauen (F) *n* = 154	*p*_Mann-Whitney-_ _U-Test M vs. F_	Gesamt *n* = 1131	Männer (M) *n* = 951	Frauen (F) *n* = 180	*p*_Mann-Whitney-_ _U-Test M vs. F_	
Beanspruchung	2,43 (0,29–5,43)	2,36 (0,29–5,43)	2,75 (0,79–5,14)	*<* *0,001*	2,86 (0,29–5,86)	2,79 (0,29–5,86)	3,21 (0,57–5,57)	**<** **0,001**	**<** **0,001**, M: ***, F: **
Allgemeine Belastung – Niedergeschlagenheit	2,50 (0–6)	2,50 (0–6)	2,50 (0–6)	*0,030*	3,00 (0–6)	3,00 (0–6)	3,50 (0–6)	**0,002**	**<** **0,001**, M: ***, F: ***
Emotionale Belastung	2,50 (0–6)	2,50 (0–6)	2,50 (0–5,5)	*0,029*	3,00 (0–6)	3,00 (0–6)	3,50 (1–6)	**0,002**	**<** **0,001**, M: ***, F: ***
Soziale Spannungen	2,50 (0–6)	2,50 (0–6)	3,0 (0,5–6)	0,081	3,00 (0–6)	3,00 (0–6)	3,50 (0,5–5,5)	**0,049**	**<** **0,001**, M: ***, F: **
Ungelöste Konflikte – Erfolglosigkeit	2,50 (0–6)	2,50 (0–6)	2,50 (0–6)	0,105	3,00 (0–6)	3,00 (0–6)	3,00 (0–6)	0,075	**0,001**, M: **, **F: n.** **s.**
Übermüdung – Zeitdruck	3,00 (0–6)	3,00 (0–6)	3,00 (0,5–5,5)	*0,019*	3,50 (0–6)	3,00 (0–6)	3,50 (0–6)	**0,008**	**<** **0,001**, M: ***, F: *
Energielosigkeit – Unkonzentriertheit	2,00 (0–5,5)	2,0 (0–5,5)	2,0 (0–5,5)	*0,008*	2,50 (0–6)	2,50 (0–6)	2,50 (0–6)	0,122	**<** **0,001**, M: ***, **F: n.** **s.**
Körperliche Beschwerden	2,00 (0–6)	2,0 (0–5,5)	2,5 (0–6)	*<* *0,001*	2,50 (0–6)	2,00 (0–6)	3,00 (0–6)	**<** **0,001**	**<** **0,001**, M: ***, **F: n.** **s.**
Erholung	2,90 (0,7–5,5)	3,00 (0,7–5,5)	2,85 (1,1–5,4)	0,207	2,60 (0,1–5,6)	2,60 (0,1–5,6)	2,50 (0,6–4,7)	0,374	**<** **0,001**, M: ***, F: **
Erfolg – Leistungsfähigkeit	3,00 (0–5,5)	3,0 (0–5,5)	3,0 (0,5–5,5)	0,217	2,50 (0–6)	2,50 (0–6)	2,50 (0,5–5)	**0,023**	**<** **0,001**, M: ***, F: **
Erholung im sozialen Bereich	3,00 (0,5–6)	3,00 (0,5–6)	3,00 (0,5–6)	0,503	2,00 (0–6)	2,00 (0–6)	2,00 (0–5)	0,358	**<** **0,001**, M: ***, F: ***
Körperliche Erholung	3,00 (0–6)	3,00 (0,5–6)	2,50 (0–5,5)	*0,016*	2,50 (0–6)	2,50 (0–6)	2,50 (0–5,5)	0,446	**<** **0,001**, M: ***, **F: n.** **s.**
Allgemeine Erholung – Wohlbefinden	3,50 (0,5–6)	3,50 (0,5–6)	3,50 (1–6)	0,951	3,00 (0–6)	3,00 (0–6)	2,50 (1–5,5)	0,453	**<** **0,001**, M: ***, F: ***
Erholsamer Schlaf	2,50 (0–6)	3,00 (0–6)	2,50 (0–6)	0,073	2,50 (0–6)	2,50 (0–6)	2,50 (0–6)	0,593	0,176, **M: n.** **s.**, **F: n.** **s***.*

Die signifikanten *p*-Werte sind fett markiert. Die Unterschiede der Mittelwertvergleiche zwischen der 1. und 2. Welle für Frauen (F) und Männer (M) sind getrennt in der letzten Spalte angegeben als *(*p* < 0,05), **(*p* < 0,01), ***(*p* < 0,001) oder n. s. (*p* > 0,05)

## Diskussion

Die Ergebnisse dieser Befragung reflektieren das Belastungserleben des Einsatzpersonals und beziehen Erholungsaktivitäten im deutschen Rettungsdienst während der ersten beiden SARS-CoV-2-Pandemie-Wellen mit ein. Damit stellen die Ergebnisse einen guten Gradmesser zur Einordnung der gegenwärtigen Situation in Bezug auf den Erholungs-Beanspruchungs-Zustand im bundesdeutschen Rettungsdienst dar. Aus der hier vorliegenden Studie geht hervor, dass die subjektive Beanspruchung seit Beginn der SARS-CoV-2-Pandemie von der ersten zur zweiten Welle zu- und die Erholung abgenommen hat.

Bereits vor der Coronapandemie galten Einsatzkräfte im Rettungsdienst als eine Berufsgruppe mit besonders hohen physischen und psychischen Belastungen im Arbeitsalltag [8, 12, 13, 17, 23]. Seit 2020 ist, bedingt durch die SARS-CoV-2-Pandemie, ein zusätzliches Belastungsempfinden für den Rettungsdienst zu verzeichnen [24].

Ausreichende Erholung und erholsamer Schlaf sind ein wesentlicher Bestandteil der physischen und psychischen Gesundheit und tragen erheblich zur allgemeinen Leistungsfähigkeit bei [5, 21]. Durch eine Beeinträchtigung der Erholungsphase kommt es zu einem veränderten psychophysischen Gesamtzustand. Eine Beanspruchung hat eine Inanspruchnahme von Ressourcen zur Folge, die sich bei steigender Belastung erschöpfen kann [23]. 91 % der befragten Rettungsdienstkräfte berichteten von einem Anstieg an Arbeitsbelastungen und verringerter Arbeitszufriedenheit im Verlauf der Pandemie [24]. Die fehlende Erholung und viele Arbeitsstunden beeinträchtigen die psychische Gesundheit des Rettungsdienstpersonals [1].

Beim Vergleich der Ergebnisse einer Studie [23] aus der Vorpandemiezeit, die das gleiche Verfahren (EBF) eingesetzt hat, fällt auf, dass die Ergebnisse in der Pandemiezeit schlechter ausfallen, d. h. geringere Werte bei der Hauptskala Erholung und höhere Werte bei der Hauptskala Beanspruchung aufweisen. Zum Vergleich lag die Beanspruchung von Einsatzkräften der Berufsfeuerwehren im Rettungsdienst schon damals außerhalb des Bereichs, der als akzeptabel zu definieren ist. So wiesen Rettungsdienstkräfte der Vorpandemiestudie bei den Berufsfeuerwehren ebenfalls eine höhere Beanspruchung und eine verringerte Erholung im EBF auf als Rettungsdienstkräfte in Hilfsorganisationen [23]. Vorpandemiestudien zu Einsatzkräften der Polizei und Bundeswehrsoldaten ergaben ein geringeres Beanspruchungsempfinden bei insgesamt gleicher oder besserer Erholungsfähigkeit in diesen Berufsgruppen [7, 11].

Die Verteilung der Geschlechter innerhalb der einzelnen Stichproben entspricht in der Gesamtbetrachtung der aktuellen Verteilung aller Einsatzkräfte im Rettungsdienst in Deutschland [26].

Da die Frauen zu unterschiedlichen Zeitpunkten der Coronapandemie eine signifikant höhere Beanspruchung (*p* < 0,001) aufwiesen, sollten die Empfehlungen im Rahmen der Prävention und Gesundheitsförderung entsprechend den Geschlechtergruppen durchgeführt werden.

### Limitationen

Als Limitation der Studie kann aufgezeigt werden, dass regionale Unterschiede je nach Ausmaß der Pandemielage nicht berücksichtigt wurden. Somit kann auch nicht sicher ausgeschlossen werden, dass besonders beanspruchte Einsatzkräfte des Rettungsdienstes überhaupt an der Studie teilgenommen haben. Als Alternativerklärung für den Unterschied sollte auch die Veränderung von Bewertungsstandards im Verlauf der Pandemie in Rechnung gestellt werden. Möglicherweise könnte eine selbst durchlaufene Coronaerkrankung oder die von Familienmitgliedern die Ergebnisse negativ beeinflussen. Diese wurde jedoch nicht erfragt. Es ist davon auszugehen, dass einige der Befragten an beiden Wellen teilgenommen haben, sodass die hier ausgewerteten 1936 Datensätze eine Obergrenze darstellen. Eine Rücklaufquote kann aufgrund der Art der Rekrutierung nicht ermittelt werden. Die Studie ist daher als nicht repräsentativ einzuschätzen, allerdings schmälert es keineswegs die Aussagekraft der Ergebnisse. Vielmehr zeigt die Studie einen wegweisenden Trend hinsichtlich zunehmender Belastung und Beanspruchung während der ersten stattgehabten Pandemiewellen.

## Fazit für die Praxis

- Die Beanspruchung und Erholung zwischen der 1. und 2. Welle haben sich in der subjektiven Bewertung verschlechtert.
- Die vorliegenden Ergebnisse verdeutlichen den notwendigen Handlungsbedarf und erfordern ein Überdenken des bisherigen Agierens zur Erhaltung der individuellen Arbeits- und Leistungsfähigkeit im Rettungsdienst.
- Die Studienlage im Rettungsdienst zu Belastungen und Beanspruchungen während der SARS-CoV-2-Pandemie ist aktuell gering bis nicht existent. Demzufolge sind weitere Studien zur Schaffung eines aussagekräftigen Forschungsstands unter Berücksichtigung der fortwirkenden Pandemielage notwendig.
- Die Entwicklung von Handlungsempfehlungen für die Praxis auf der Grundlage von Studienergebnissen ist sinnvoll. Darüber hinaus ist es wichtig, geschlechtergruppenspezifische Unterschiede zu beachten, um der höheren Beanspruchung durch die Stärkung von Ressourcen entgegenzuwirken.
- Die Beachtung des Erholungs- und Belastungszustands der Einsatzkräfte zur Aufrechterhaltung der physischen und psychischen Gesundheit ist ein wichtiger Bestandteil für eine gesunde und gelingende Organisation im Rettungsdienst. Diese kann im Rahmen der Gefährdungsbeurteilung im Rettungsdienst genutzt werden und entspricht der Fürsorgepflicht des Arbeitgebers nach dem Arbeitsschutzgesetz.
- Die interdisziplinäre Vernetzung zu anderen Gesundheitsprofessionen erscheint sinnvoll, da die Studienlage mitunter umfangreicher und differenzierter ist als im Rettungsdienst. Ein Erfahrungsaustausch kann hier neue Perspektiven und Lösungsansätze generieren.
